# A high-throughput screening platform for acetylcholinesterase inhibitors using a genetically encoded acetylcholine fluorescent sensor

**DOI:** 10.3389/fbioe.2026.1781867

**Published:** 2026-02-27

**Authors:** Xinxin Li, Yueming Yu, Siyu Li, Xueyang Lin, Chen Yang, Yufeng Yang, Shengran Wang, Mengwei Zhou, Zhenghao Bao, Xin Sui, Wenya Feng, Jun Yang, Daiying Zuo, Yuan Luo, Yongan Wang, Xianli Du

**Affiliations:** 1 Department of Pharmacology, Shenyang Pharmaceutical University, Shenyang, China; 2 Academy of Military Medical Sciences, Beijing, China

**Keywords:** AChE, GRAB probe, high-throughput screening, inhibitor, stable cell line

## Abstract

Acetylcholinesterase (AChE) is a crucial hydrolytic enzyme in the central nervous system, responsible for the rapid degradation of the neurotransmitter acetylcholine (ACh) in the synaptic cleft, thereby maintaining the balance between neuronal excitation and inhibition. AChE is not only the primary target of neurotoxic agents and organophosphorus pesticides but its aberrant activity is also closely associated with various neurodegenerative diseases such as Alzheimer’s disease (AD) and myasthenia gravis. The efficient and rapid discovery and screening of AChE inhibitors hold urgent and significant value for chemical toxin detection, toxicological research, and drug development for neurodegenerative diseases. Addressing the limitations of existing methods, such as low biocompatibility, low detection throughput, relative operational complexity, and high cost, this study innovatively utilizes a genetically encoded biosensor to construct a stable cell line co-expressing the ACh probe and AChE, establishing a novel high-throughput screening method for AChE inhibitors. The results demonstrate that this method achieved to detect AChE inhibitors at micromole level. This method eliminates the need for purified enzymes and toxic chemical reagents (e.g., DTNB in Ellman’s assay), significantly reduces cost (by approximately two orders of magnitude), and offers a simplified, rapid, and high-throughput compatible workflow for applications in neurotoxin detection and neurotherapeutic drug discovery.

## 1 Introduction

Acetylcholinesterase (AChE) is the key enzyme catalyzing acetylcholine hydrolysis in the central nervous system and serves as the primary target for neurotoxic agents and organophosphorus pesticides (Kumar et al., 2023; Schwenk, 2018). Inhibiting AChE activity leads to excessive accumulation of ACh, causing overstimulation of muscarinic and nicotinic receptors in the central and peripheral nervous systems, which result in severe toxic reactions or even death (Kumar et al., 2023; Schwenk, 2018). Presently, substantial amounts of organophosphates continue to be released into the environment, persistently contaminating ecosystems, the food chain, and water sources. Even at extremely low concentrations, these toxins pose a serious threat to human health and public safety (Li H. et al., 2023). Chronic exposure may induce neurodevelopmental disorders, cardiovascular damage, and gene mutations through bioaccumulation. Additionally, artificial intelligence (AI) technologies for drug discovery could be misused for *de novo* design of toxins (Urbina et al., 2022). The activity of numerous compounds needs to be address for public safety. Furthermore, abnormal AChE activity is closely linked to neurodegenerative diseases including Alzheimer’s disease (AD) and myasthenia gravis. Studies indicate that inhibiting AChE activity can effectively increase synaptic ACh concentration, thereby improving cognitive dysfunction in AD patients (Gao et al., 2021). Consequently, the rapid assessment of AChE inhibitory activity is of great significance for toxin detection and the discovery of neurotherapeutic agents.

Researchers have extensively explored methods for detecting AChE inhibitory activity (Tan et al., 2024; Ding et al., 2023; Gong et al., 2022; Meng et al., 2023). Traditional spectrophotometric methods [e.g., Ellman et al. (1961)], which rely on chromogenic reactions with thiocholine substrates, offer simplicity but are susceptible to sample background interference, exhibit low sensitivity, and often employ reagents like DTNB that are notably toxic. Chromatographic techniques (e.g., HPLC, GC/LC-MS), while significantly improving precision, involve high instrument costs, complex sample pretreatment, time-consuming analysis, and require specialized operators, making them unsuitable for high-throughput screening (HTS) demands (Gong et al., 2022). Immunoassays (e.g., Enzyme-Linked Immunosorbent Assay, ELISA) are limited by factors such as antibody cross-reactivity, poor thermal stability, and high development costs. In the realm of novel sensing technologies, photoelectrochemical sensors based on nanomaterials (Tan et al., 2024; Liang et al., 2013; Li G. et al., 2023) (e.g., gold nanoparticles, quantum dots) have achieved pM-level detection via signal amplification strategies but suffer from insufficient stability. Microfluidic chip technology enhances detection efficiency through integrated design but is constrained by complex fabrication processes (Pundir et al., 2019). These emerging methods exhibit strong dependence on specialized equipment and have yet to achieve widespread application.

GPCR-activation-based sensors (GRAB) are a class of recently developed genetically encoded biosensors that convert ligand-induced conformational changes of G protein-coupled receptors (GPCRs) into fluorescence signals via an intramolecular circularly permuted green fluorescent protein (cpGFP). This design enables real-time, *in situ* detection of a series of neurotransmitters including acetylcholine (Jing et al., 2018), dopamine (Sun et al., 2018), serotonin (Deng et al., 2024), norepinephrine (Feng et al., 2024), and neuropeptides (Wang et al., 2023). Based on this principle, this study proposes a novel method for assessing AChE inhibitory activity utilizing an acetylcholine GRAB sensor. The method involves co-expressing AChE and the GRAB probe in HEK293T cells and using the GRAB sensor to directly detect ACh levels in the reaction system, thereby rapidly evaluating the inhibitory activity of test samples against AChE. Compared to colorimetric methods like the Ellman assay, the method described herein does not rely on reactions between enzymatically generated thiocholine and auxiliary reagents, avoiding the use of toxic and unstable chemicals like DTNB. More importantly, since purified acetylcholinesterase is no longer required, the screening cost is reduced by two orders of magnitude. With its operational convenience, this method presents an optimal approach for screening organophosphorus agent samples and for the high-throughput screening of novel AChE inhibitors.

## 2 Materials and methods

### 2.1 Cell culture

HEK293T cell lines were purchased from Cell resource center, IBMS, and cultured at 37 °C, 5% CO_2_, and 95% air in DMEM (Dulbecco’s Modified Eagle Medium containing 4.5 g glucose/mL; 11965092, Gibco) supplemented with 1% Penicillin/Streptomycin (CC004, MACGENE) and 10% Fetal Bovine Serum (FBS; 10099141C, Gibco). This culture medium was used for all experiments.

### 2.2 Common reagents and antibodies

The following reagents were purchased from commercial sources: puromycin (R23002, YUANYE Inc), G418 disulfate salt (A1720, Sigma), Lipofectamine™ 3000 transfection kit (L3000015, Invitrogen), Opti-MEM™ (31985070, Gibco), calcium phosphate transfection kit (CTK001, MACGENE), PrimeSTAR® HS DNA Polymerase (R010A, Takara), NEBuilder HiFi DNA Assembly Master Mix (E2621L, NEB), Acetylcholine chloride (249495, J&K), Neostigmine Bromide (N838484, Macklin), Physostigmine (P922786, Macklin), dimethyl sulfoxide (DMSO, D2650, Sigma), aspirin (A2093, Sigma), serotonin (S31021, YUANYE), dimethoate (D109819, Aladdin), chlorpyrifos (C109843, Aladdin), Donepezil Hydrochloride (D849374, Macklin).

Antibodies were as follows: Goat anti-Rabbit IgG (H + L) Cross-Adsorbed Secondary Antibody (A11012, Invitrogen), Rabbit anti-Flag Antibody (14793T, Sigma), polyclonal rabbit anti-Flag (F1804, Sigma), Anti-rabbit IgG, HRP-linked Antibody (7074, CST), Cy3-conjugated goat anti-mouse (A0521, Beyotime).

### 2.3 DNA constructs

The expression cassette of ACh3.0-IRES-mcherry-CAAX was amplified from the pDisplay-Ach3.0 plasmids from Li Lab (Jing et al., 2020) and inserted into the pCDH-CMV-MCS-EF1-puro lentiviral vector between the XbaI and EcoRI restriction sites, to generate the pCDH-ACh-puro plasmid. The AChE gene (Genbank: NM_000665) with 3×Flag Tag in the 3′ terminal was amplified from a cloning vector purchased from Youbio (G152420) and inserted into GV350 lentiviral vector (GeneChem) to generate the Ubc-ACHE-3Flag-SV40-Neomycin plasmid. All the constructs were confirmed by DNA sequencing. Lentivirus was obtained following the standard three-plasmid packaging protocol.

### 2.4 RT-qPCR analysis

Total RNA was isolated from cultured cells using the RNeasy Kit (QIAGEN). cDNA was synthesized by reverse transcription conducted with the PrimeScript™ RT Kit (Takara Bio) under programmed thermal cycling: 37 °C (15 min), 85 °C (5 s), and 4 °C. Gene expression was quantified on a Bio-Rad CFX-96 thermocycler using SYBR Green PCR-Mix (Takara Bio). The ddCt method was used to calculate the relative change of gene expressions. Quantitation of target gene was normalized to GAPDH.

The primer sequences for qRT-PCR analysis are as followed:

CAAX-qF: GGT​ACT​GCT​GCT​CTG​GGT​TC.

CAAX-qR: GCT​GCT​CGA​GAA​ACA​TTG​TAG​C.

GAPDH-qF: CGT​CAT​GGG​TGT​GAA​CCA​TG.

GAPDH-qR: GGA​CTG​TGG​TCA​TGA​GTC​CT.

### 2.5 Western immunoblotting

For protein extraction, cultured HEK293T cells infected with indicated lentivirus were collected and lysed in RIPA buffer containing 20 mM Tris–HCI pH 7.5, 150 mM NaCl, 10 mM EDTA, 1% Triton X-100, 1 mM phenylmethylsulfonyl fluoride (PMSF), and complete protease inhibitor. Cell lysates were sonicated and centrifuged at 12000 rpm. The protein concentration of each sample was determined with a BCA assay kit, following the manufacturer’s instructions. Equal amounts of protein were separated by SDS-PAGE on 8%–12% polyacrylamide gels and subsequently transferred onto PVDF membranes. The membranes were blocked with 5% non-fat milk in Tris-buffered saline containing 0.1% Tween-20 (TBST) for 1 h at room temperature, followed by incubation with specific primary antibodies overnight at 4 °C. After washing three times with TBST, the membranes were incubated with appropriate horseradish peroxidase (HRP)-conjugated secondary antibodies for 1 h at room temperature. Protein bands were visualized using an enhanced chemiluminescence (ECL) detection system and imaged with a chemiluminescence imaging system (Gel DocTM XR + imaging system, Bio-Rad).

### 2.6 Immunofluorescence staining

The HEK293T cell line that stably expresses GACh3.0 and AChE (human), were plated in a 24-well tissue culture plate at a density of 5 × 10^4^ cells/well and incubated overnight. Cells were fixed in 4% paraformaldehyde for 20 min at room temperature, washed with PBS and then blocked with blocking buffer for 30 min at room temperature, followed by incubation with primary antibody anti-Flag antibody (1:500, polyclonal rabbit anti-Flag, Sigma, F1804) overnight at 4 °C. Next day, wash the primary antibody with PBS, cells were incubation with secondary antibody labeled with Cy3-conjugated goat anti-mouse (1:500, Beyotime, A0521) for 1 h at room temperature in the dark. After thorough washing with PBS, 2 drops per well nuclear dye (Hoechst 33342, 1:2,000, Invitrogen) for 1 h at room temperature in the dark, adding liquid antifade and images were obtained with the confocal microscope (Nikon AX), the acquisition software was NIS-Elements V5.24.01.

### 2.7 Fluorescence imaging

The detection of the fluorescence changes of GACh3.0 was performed as previously described (Jing et al., 2020). Briefly, cultured HEK293T cells expressing the GACh3.0 sensor were first imaged using the Opera Phenix High-Content Screening System (PerkinElmer) equipped with a ×20/1.15-NA water-immersion objective, a 488-nm laser and a 561-nm laser; the GACh3.0 sensor’s signal was obtained using a 525/50-nm emission filter, and the mCherry–CAAX signal was obtained using a 600/30-nm emission filter. The ratio between green (G) and red (R) fluorescence was calculated before and after application of 100 μM ACh, and the change in the G/R ratio was used as the fluorescence response.

### 2.8 AChE inhibitor screening

HEK293T cells stably expressing GACh3.0 and AChE (human) were plated in a 96-well tissue culture plate at a density of 1 × 10^4^ cells/well and incubated overnight. Test compounds were added to the cell culture medium at a 1:100 ratio to achieve a final concentration of 10 μM, followed by incubation at 37 °C for 5 min. Subsequently, 100 μM acetylcholine was added to each well, and incubation continued at 37 °C for 2 min. The 96-well plate was then placed into the Opera Phenix High-Content Screening System (PerkinElmer) to detect fluorescence intensity in different samples, following the method described in Section 2.7. If a compound effectively inhibits AChE activity, a significant increase in fluorescence intensity will be detected in that sample group.

### 2.9 Statistical analysis

Statistical analyses were performed with a two-tailed unpaired t-test or as indicated in the legends. P values are indicated by asterisks in the figures as followed: *p < 0.05, **p < 0.01, ***p < 0.001 and ****p < 0.0001. Differences with a P value of 0.05 or less were considered significant. Data visualization was conducted using GraphPad Prism 10.

## 3 Results

### 3.1 Design of a GRAB-based screening method for AChE inhibitory activity

**FIGURE 1:**
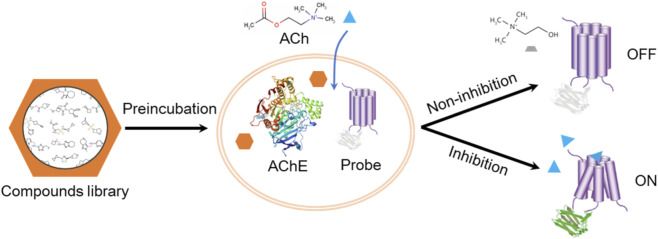
Schematic illustration of the screening principle for AChE inhibitors based on a genetically encoded probe.

This study aimed to design a novel, low-cost, high-throughput method for screening AChE activity by leveraging the specific recognition of acetylcholine by a genetically encoded biosensor. First, a stable cell line co-expressing the fluorescent probe and AChE was constructed. During drug screening, the cells are first co-incubated with the test compound, followed by the addition of a specific concentration of ACh. If the compound inhibits AChE activity, the ACh present in the system will induce a significant upregulation of the fluorescent probe signal. Conversely, if the test compound lacks inhibitory activity, the ACh will be rapidly hydrolyzed by the cell-expressed AChE, resulting in no significant change in the detected fluorescence signal. A schematic diagram of this principle is shown in Figure 1.

### 3.2 Stable expression of the acetylcholine GRAB probe in HEK293T cells

**FIGURE 2:**
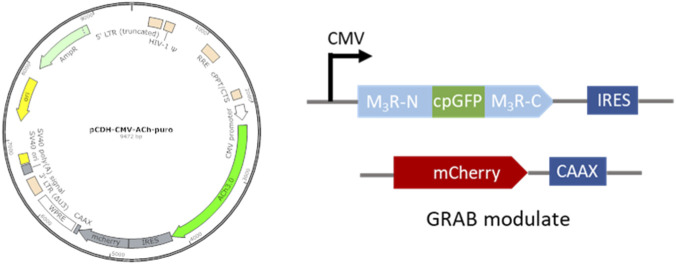
Lentiviral transfer plasmid carrying the GACh3.0 coding sequence.

The GRAB probe is a genetically encoded fluorescent sensor that can be efficiently expressed on the cell membrane. In this study, the acetylcholine probe GACh3.0, developed by Li Lab (Jing et al., 2020), was cloned into a lentiviral vector to produce GACh3.0 lentivirus. GACh3.0 primarily consists of two modules: the muscarinic receptor M3R (a natural receptor for ACh sensing) with an inserted cpGFP, and the red fluorescent protein mCherry carrying a CAAX membrane localization sequence. mCherry serves as an internal reference for fluorescence intensity to accurately measure changes in green fluorescence. These modules were integrated into a lentiviral transfer plasmid (Figure 2). The transfer plasmid and helper plasmids were transfected into HEK293T cells using the calcium phosphate method, and virus was collected after 48 h. The titer of the concentrated virus reached 2 × 10^8^ TU/mL.

**FIGURE 3:**
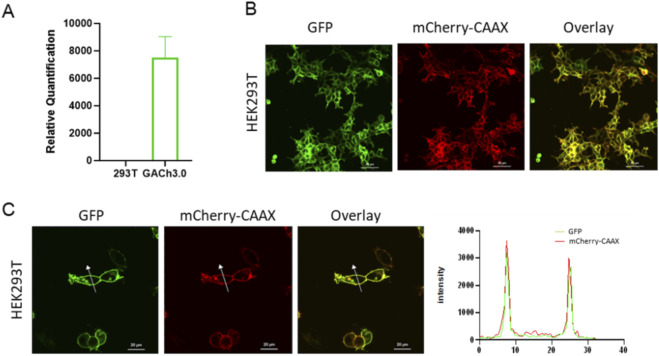
Membrane expression of GACh3.0 in HEK293T cells. **(A)** RT-qPCR verification of the translation of GACh3.0 in HEK293T cells. **(B)** Spectroscopy of co-expressed of cpGFP and mCherry in HEK293T cells. **(C)** Membrane location of GACh3.0.

Subsequently, HEK293T cells were infected with the virus at an MOI of 5, and stable HEK293T cell lines expressing GACh3.0 (GACh-Lenti) were obtained via puromycin selection. Expression of the target gene post-lentiviral infection was assessed by quantitative PCR (qPCR) and confocal microscopy. qPCR results showed that the relative quantification (RQ) value of the target gene in lentivirus-infected 293T cells reached 7420.25 compared to wild-type 293T cells, indicating high-efficiency probe expression (Figure 3A). Confocal microscopy results (Figure 3B) revealed significant expression of both green and red fluorescent proteins 48 h post-infection. Both GACh3.0 and mCherry-CAAX exhibited proper membrane localization in 293T cells (Figure 3C). These results demonstrate that the constructed stable cell line achieves efficient and active expression of GACh3.0.

### 3.3 Active expression of AChE in HEK293T cells

**FIGURE 4:**
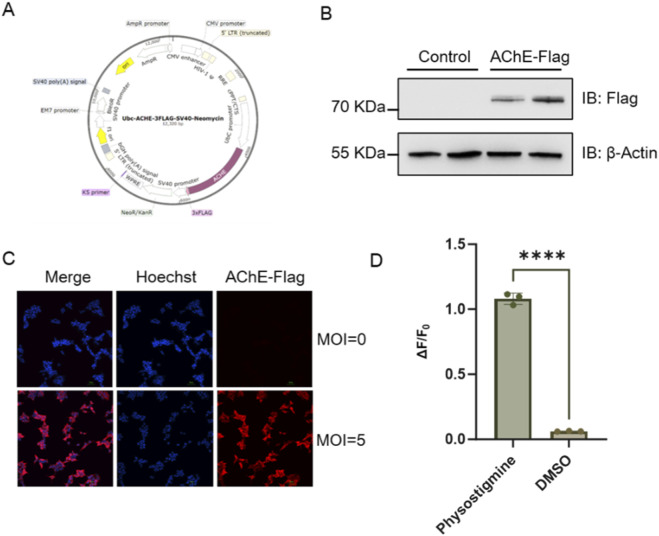
Active Expression of AChE in HEK293T Cells. **(A)** The lentiviral vector for expression of AChE. **(B)** Western blotting of AChE with anti-Flag antibody in AARC cells. **(C)** Immunofluorescence of AChE with anti-Flag antibody and Cy3-conjugated second antibody in AARC cells. MOI indicates the multiplicity of AChE virus infection. **(D)** The fluorescence response (ΔF/F_0_) of AARC cell in response to 100 μM ACh with or without 10 μM Physostigmine (DMSO solvent control group).

To achieve stable AChE expression in the aforementioned cells, we then constructed another lentiviral plasmid for AChE (Figure 4A), and produced lentiviral particles with a concentrated viral titer 1.4 × 10^9^ TU/mL. The GACh-Lenti cells stably expressing GACh3.0 were infected with the prepared lentivirus to achieve co-expression of AChE and GACh3.0. Polyclonal stable cell lines were selected using puromycin and G418, yielding AARC cells (Acetylcholinesterase Activity Reporter Cells). The expression of AChE in AARC cells was verified by Western blotting (Figure 4B) and Immunofluorescence (Figure 4C).

Furthermore, to verify whether the AChE expressed in the cell line possessed normal enzymatic activity, AARC cells were pre-treated with 10 μM physostigmine (acetylcholinesterase inhibitor) or DMSO solvent for 5 min. Then 100 μM acetylcholine was added to the culture medium. It was observed that acetylcholine in the cells without physostigmine was rapidly hydrolyzed, resulting in a fluorescence intensity significantly lower than that of the DMSO control group (Figure 4D). This result indicates that the AChE expressed in the cells exhibits robust hydrolytic activity, demonstrating that the functionality of the co-expressing cell line aligns with our design expectations.

### 3.4 Optimization and testing of the screening system

**FIGURE 5:**
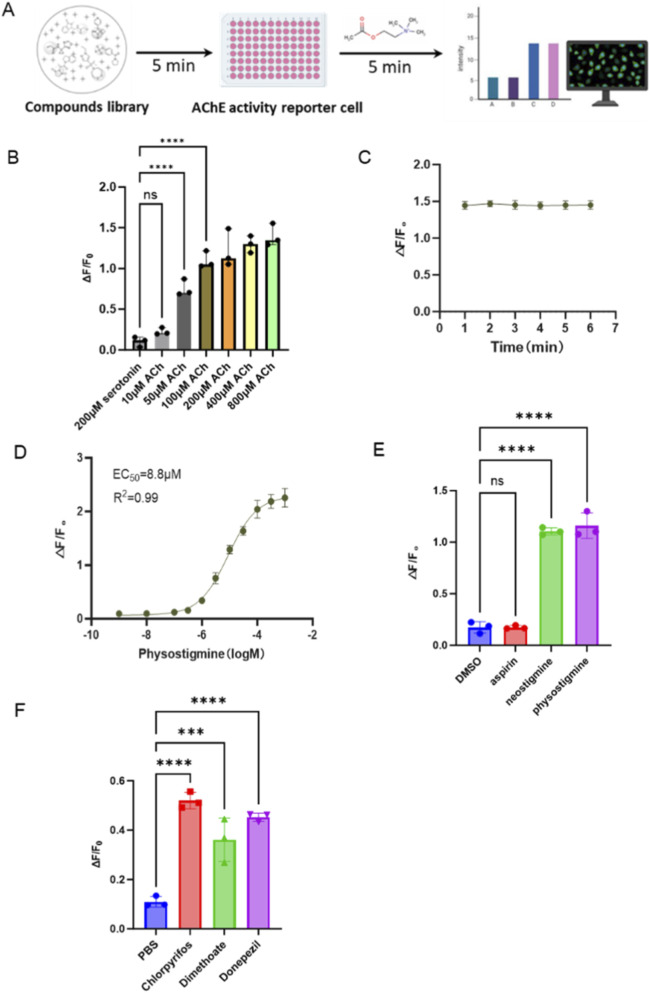
Optimization and testing of screening conditions for AChE inhibitors based on AARC cells. **(A)** Workflow of the Acetylcholinesterase Inhibitor High-Throughput Screening Process. **(B)** The fluorescence response (ΔF/F_0_) of AARC cell in response to different concentration of ACh with neostigmine. serotonin was set as a non-relevant ligand control. **(C)** The fluorescence signal continuous for more than 7 min after acetylcholine addition. **(D)** EC_50_ value for physostigmine in this screening methods. **(E,F)** The fluorescence response (ΔF/F_0_) of AARC cell in response to indicated chemicals, DMSO and PBS were set as solvent control group, respectively.

After achieving stable activity expression of GACh3.0 and AChE in AARC cells, we designed an acetylcholinesterase inhibitor screening platform based on AARC cells, as shown in Figure 5A. First, the test compounds were co-incubated with the cells for 5 min to ensure sufficient binding between the compounds and the targets. Then acetylcholine was added to detect the activation of GACh3.0. Due to the rapid hydrolysis of acetylcholine catalyzed by acetylcholinesterase, a significant change in fluorescence signal intensity could be observed shortly after the addition of acetylcholine.

We next tested and optimized both the concentration of acetylcholine added and the duration of the signal. Considering the high hydrolytic activity of AChE towards ACh, the ACh concentration needed to be set at an appropriate level. If too low, ACh would be easily hydrolyzed, making signal detection difficult; if too high, it could lead to false positives. Given that the EC_50_ of the GACh3.0 probe’s response to ACh is at the micromolar level (Pundir et al., 2019), (Jing et al., 2020), we tested six concentration gradients (10 μM, 50 μM, 100 μM, 200 μM, 400 μM, and 800 μM), using serotonin (inactive on GACh3.0) as a control, to assess the cellular fluorescence response. To simulate chemical toxin inhibition of AChE, 100 μM neostigmine (an AChE inhibitor) was pre-added to the cell culture medium. The results (Figure 5B) showed that 50 μM ACh was sufficient to achieve a highly significant difference (P < 0.001). Considering a wider Screening window is needed to meet the requirements for screening compounds with varying levels of AChE inhibitory activity, 100 μM ACh was selected for standard protocol. Regarding signal duration, we monitored the fluorescence signal intensity continuously for 7 min after acetylcholine addition and observed no significant signal decay (Figure 5C). This timeframe is sufficient to complete the scanning of an entire 96-well plate.

The final optimized system requires less than 10 minutes to complete a batch detection (using 96-well or 384-well plates), with an average detection cost of 0.86 RMB per sample (see the cost breakdown in Supplementary Table S1). Compared to an existing commercial AChE activity assay kits (e.g., Sigma, MAK119), this represents a cost reduction of approximately two orders of magnitude.

To further validate the sensitivity of the screening system, we tested two AChE inhibitors, neostigmine bromide (100 μM) and physostigmine (10 μM). Aspirin was set as the negative control which has no inhibitory activity on AChE. Compared to the inactive aspirin and solvent control groups, both inhibitor compounds showed significant differences on the fluorescence signal (Figure 5E). In terms of sensitivity, the results indicated an EC_50_ value for physostigmine of approximately 8.8 μM, which generally meets the requirements for compound high-throughput screening (Figure 5D).

Next the inhibitory activities of two pesticides, dimethoate and chlorpyrifos, and an approved drug for Althamer’s, donepezil hydrochloride, were evaluated by the screening method. AARC cells were pretreated these three chemicals (20 μM) for 5 min, followed by the addition of ACh with centration of 100 μM. Since these compounds are water-soluble, we employed Phosphate buffer saline (PBS) as the solvent and vehicle control. The results showed that all these chemicals induced significant fluorescence signal increase (Figure 5F), indicating that our screening methods is suitable for the test of different kinds of compounds.

## 4 Discussion

Acetylcholinesterase (AChE) is a crucial hydrolytic enzyme in the cholinergic system, responsible for the rapid degradation of the neurotransmitter acetylcholine (ACh) in the synaptic cleft. This process precisely terminates cholinergic signaling, maintaining the balance between neuronal excitation and inhibition. The precise regulation of its activity is decisive for numerous physiological processes, including learning, memory, muscle contraction, and autonomic function. Therefore, the stability of AChE functional state is fundamental for the normal operation of the central and peripheral nervous systems.

Given the central role of AChE in neuronal signaling, it serves as the target for various neurotoxic agents (e.g., organophosphates and carbamates) (Wang X. et al., 2022). These agents cause irreversible or reversible inhibition of AChE activity, leading to massive ACh accumulation in the synaptic cleft and triggering a cholinergic crisis. On the other hand, in the pathological progression of neurodegenerative diseases like Alzheimer’s disease, dysfunction of cholinergic neurons is closely associated with cognitive decline. Strategies to elevate synaptic ACh levels, particularly through the use of reversible AChE inhibitors, have become first-line clinical treatments for symptom relief (Zhao et al., 2024; Saud et al., 2024; Saxena and Dubey, 2019). Consequently, the efficient and rapid discovery and screening of novel, highly selective, low-toxicity AChE modulators (especially inhibitors) hold urgent and significant value for toxicological research, the development of neurotoxic agent antidotes, and drug discovery for neurodegenerative diseases.

In recent years, new methods for detecting AChE activity continue to be explored, which can be broadly categorized by their experimental principles as follows: biochemical colorimetry, novel nanoprobes, and computational virtual screening. The colorimetric method based on Ellman’s reagent and its fluorescent variants remain the most widely used (Wang Y. et al., 2022; Zavala-Ocampo et al., 2022), with their core advantages being technical maturity, operational standardization, and high-throughput compatibility. However, these methods typically rely on extracted enzymes or tissue homogenates, making it difficult to reflect compound effects in real-time within living cell systems that more closely mimic the physiological environment. More importantly, they generally require chemical chromogenic agents such as 5,5′-dithiobis(2-nitrobenzoic acid) (DTNB), some components of which may possess cytotoxicity or chemical instability. This not only increases operational risks and complexity but may also interfere with the activity assessment of certain test compounds. Furthermore, these methods have room for improvement in terms of throughput, cost, and suitability for miniaturized, automated high-throughput screening (HTS) platforms. In contrast, the emerging nanosensing methods in recent years (e.g., those based on metal-organic frameworks (Li et al., 2024), lanthanide hybrid materials (Zhang et al., 2024), or plasmonic nanoparticle sensors (Cai et al., 2025)) represent a significant technological advancement. Through ingenious nanostructure design, they achieve signal amplification, significantly enhancing detection sensitivity, making them particularly suitable for point-of-care testing (POCT) and high-sensitivity analytical needs.

However, whether traditional colorimetric methods or novel nanosensors, their fundamental paradigm remains *in vitro* biochemical reactions. They typically treat AChE as an isolated biomolecule for activity measurement in a preset buffer system. Although virtual screening methods (Xu et al., 2023) can predict compound binding to the enzyme’s active site *in silico* with unprecedented throughput, greatly accelerating lead compound discovery, their results ultimately require validation through *in vitro* enzymatic assays. Therefore, these methods share a core limitation: difficulty in reflecting the true pharmacodynamic behavior of inhibitors within intact living cells, including compound cell membrane permeability, metabolic stability intracellularly, and inhibitory selectivity against actively expressed AChE within cells.

Addressing the aforementioned challenges, this study developed and established a novel screening method for AChE activity modulators based on genetically encoded biosensors, which is essentially a functional analysis performed in cellulo (within living cells). This method involves the stable genomic integration of genes encoding both AChE and an ACh probe into host cells, thereby constructing a sustainable, homogenously responsive “sensor cell line.” The superiority of this method is mainly reflected in the following aspects: First, it dispenses with the toxic or unstable chemical auxiliaries used in traditional methods. The entire detection system requires only the ACh substrate and cells stably co-expressing the biosensor and AChE. The system is simple, highly biocompatible, significantly reducing potential safety hazards and chemical interference with detection results. Second, the method offers significant cost-effectiveness. Preliminary calculations indicate that its reagent cost per single assay can be reduced by approximately two orders of magnitude compared to the traditional Ellman method, making large-scale screening projects highly economically feasible. Finally, being cell-based, the method is easily adaptable to multi-well plate formats with straightforward operational procedures, making it highly suitable for automated, high-throughput primary drug screening. It provides a powerful tool for rapidly discovering lead compounds from vast compound libraries.

It is noteworthy that the method established in this study also possesses strong extensibility. On one hand, the high-throughput screening method proposed in this paper is primarily intended for the first-round primary screening of a large number of candidate compounds, the incubation time was set to 5 min to enhance screening throughput. Researchers can perform long-term, continuous data acquisition on the same population of cells, thereby obtaining precise kinetic parameters of inhibitor action, such as onset time, inhibition duration, and recovery period. This time-resolved information, unavailable from *in vitro* endpoint assays, is crucial for evaluating the duration of drug action (e.g., distinguishing reversible from irreversible inhibitors). On the other hand, both butyrylcholinesterase (BChE) and AChE are therapeutic targets for Alzheimer’s disease (Darvesh, 2016; Geula and Darvesh, 2004) and both possess the ability to hydrolyze ACh. Following the rationale developed in this study, one could construct a cell line stably co-expressing BChE and the GACh3.0 sensor to enable screening for BChE activity modulators. Considering the different efficiency of acetylcholine hydrolysis between BChE and AChE, the concentration of acetylcholine added and the reaction time would both require further optimization.

In summary, the novel screening method established in this study, with its safety, low cost, high compatibility, and extensibility potential, offers a competitive alternative for the discovery of AChE modulators. It holds promise for accelerating the research and development process of related therapeutic agents and tool compounds.

## Data Availability

The original contributions presented in the study are included in the article/Supplementary Material, further inquiries can be directed to the corresponding authors.

## References

[B1] Cai Y. Li Y. Wang Y. Xu Y. Chen T. Xue R. (2025). Triple-mode sensing platform for acetylcholinesterase activity monitoring and anti-Alzheimer's drug screening based on a highly stable Cu (I) compound. Biosens. Bioelectron. 271, 117078. 10.1016/j.bios.2024.117078 39708491

[B2] Darvesh S. (2016). Butyrylcholinesterase as a diagnostic and therapeutic target for Alzheimer's disease. Curr. Alzheimer Res. 13 (10), 1173–1177. 10.2174/1567205013666160404120542 27040140

[B3] Deng F. Wan J. Li G. Dong H. Xia X. Wang Y. (2024). Improved green and red GRAB sensors for monitoring spatiotemporal serotonin release *in vivo* . Nat. Methods 21 (4), 692–702. 10.1038/s41592-024-02188-8 38443508 PMC11377854

[B4] Ding R. Li Z. Xiong Y. Wu W. Yang Q. Hou X. (2023). Electrochemical (bio)Sensors for the detection of organophosphorus pesticides based on nanomaterial-modified electrodes: a review. Crit. Rev. Anal. Chem. 53 (8), 1766–1791. 10.1080/10408347.2022.2041391 35235478

[B5] Ellman G. L. Courtney K. D. Andres V. Jr. Feather-Stone R. M. (1961). A new and rapid colorimetric determination of acetylcholinesterase activity. Biochem. Pharmacol. 7, 88–95. 10.1016/0006-2952(61)90145-9 13726518

[B6] Feng J. Dong H. Lischinsky J. E. Zhou J. Deng F. Zhuang C. (2024). Monitoring norepinephrine release *in vivo* using next-generation GRAB(NE) sensors. Neuron 112 (12), 1930–1942.e6. 10.1016/j.neuron.2024.03.001 38547869 PMC11364517

[B7] Gao H. Jiang Y. Zhan J. Sun Y. (2021). Pharmacophore-based drug design of AChE and BChE dual inhibitors as potential anti-alzheimer's disease agents. Bioorg Chem. 114, 105149. 10.1016/j.bioorg.2021.105149 34252860

[B8] Geula C. Darvesh S. (2004). Butyrylcholinesterase, cholinergic neurotransmission and the pathology of Alzheimer's disease. Drugs Today (Barc) 40 (8), 711–721. 10.1358/dot.2004.40.8.850473 15510242

[B9] Gong C. Fan Y. Zhao H. (2022). Recent advances and perspectives of enzyme-based optical biosensing for organophosphorus pesticides detection. Talanta 240, 123145. 10.1016/j.talanta.2021.123145 34968808

[B11] Jing M. Zhang P. Wang G. Feng J. Mesik L. Zeng J. (2018). A genetically encoded fluorescent acetylcholine indicator for *in vitro* and *in vivo* studies. Nat. Biotechnology 36 (8), 726–737. 10.1038/nbt.4184 29985477 PMC6093211

[B12] Jing M. Li Y. Zeng J. Huang P. Skirzewski M. Kljakic O. (2020). An optimized acetylcholine sensor for monitoring *in vivo* cholinergic activity. Nat. Methods 17 (11), 1139–1146. 10.1038/s41592-020-0953-2 32989318 PMC7606762

[B13] Kumar V. Kim H. Pandey B. James T. D. Yoon J. Anslyn E. V. (2023). Recent advances in fluorescent and colorimetric chemosensors for the detection of chemical warfare agents: a legacy of the 21st century. Chem. Soc. Rev. 52 (2), 663–704. 10.1039/d2cs00651k 36546880

[B14] Li H. Jiao Y. Li L. Jiao X. (2023a). Research progress and trend of effects of organophosphorus pesticides on aquatic organisms in the past decade. Comp. Biochem. Physiol. C Toxicol. Pharmacol. 271, 109673. 10.1016/j.cbpc.2023.109673 37268167

[B15] Li G. Huang X. Peng C. Sun F. (2023b). Highly sensitive fluorescence detection of three organophosphorus pesticides based on highly bright DNA-templated silver nanoclusters. Biosens. (Basel) 13 (5), 520. 10.3390/bios13050520 37232881 PMC10216281

[B16] Li Y. Chen L. Li C. Y. Zhang J. Zhao Y. Yang Y. H. (2024). Nanoplasmonic biosensors for multicolor visual analysis of acetylcholinesterase activity and drug inhibitor screening in point-of-care testing. Biosens. Bioelectron. 247, 115912. 10.1016/j.bios.2023.115912 38096721

[B17] Liang M. Fan K. Pan Y. Jiang H. Wang F. Yang D. (2013). Fe3O4 magnetic nanoparticle peroxidase mimetic-based colorimetric assay for the rapid detection of organophosphorus pesticide and nerve agent. Anal. Chem. 85 (1), 308–312. 10.1021/ac302781r 23153113

[B18] Meng W. Q. Sedgwick A. C. Kwon N. Sun M. Xiao K. He X. P. (2023). Fluorescent probes for the detection of chemical warfare agents. Chem. Soc. Rev. 52 (2), 601–662. 10.1039/d2cs00650b 36149439

[B19] Pundir C. S. Malik A. Preety (2019). Preety, Bio-sensing of organophosphorus pesticides: a review. Biosens. Bioelectron. 140, 111348. 10.1016/j.bios.2019.111348 31153016

[B20] Saxena M. Dubey R. (2019). Target enzyme in Alzheimer's disease: Acetylcholinesterase inhibitors. Curr. Top. Med. Chem. 19 (4), 264–275. 10.2174/1568026619666190128125912 30706815

[B21] Saud A. Krishnaraju V. Taha A. Kalpana K. Malarkodi V. Durgaramani S. (2024). Potential acetylcholinesterase inhibitors to treat Alzheimer's disease. Eur. Rev. Med. Pharmacol. Sci. 28 (6), 2522–2537. 10.26355/eurrev_202403_35759 38567612

[B22] Schwenk M. (2018). Chemical warfare agents. Classes and targets. Toxicol. Lett. 293, 253–263. 10.1016/j.toxlet.2017.11.040 29197625

[B23] Sun F. Zeng J. Jing M. Zhou J. Feng J. Owen S. F. (2018). A genetically encoded fluorescent sensor enables rapid and specific detection of dopamine in flies, fish, and mice. Cell 174 (2), 481–496.e19. 10.1016/j.cell.2018.06.042 30007419 PMC6092020

[B24] Tan X. Yu C. Tang J. Wu W. Yang Q. Hou X. (2024). Progress in nanomaterials-based enzyme and aptamer biosensor for the detection of organophosphorus pesticides. Crit. Rev. Anal. Chem. 54 (2), 247–268. 10.1080/10408347.2022.2072678 35549956

[B25] Urbina F. Lentzos F. Invernizzi C. Ekins S. (2022). Dual use of artificial intelligence-powered drug discovery. Nat. Mach. Intell. 4 (3), 189–191. 10.1038/s42256-022-00465-9 36211133 PMC9544280

[B26] Wang X. Wang X. Feng R. Fu T. Jiang Y. Zhang J. (2022a). Recent advances of chemosensors for nerve agents. Chem. Asian J. 17 (14), e202200284. 10.1002/asia.202200284 35599234

[B27] Wang Y. Xue Y. Zhao Q. Wang S. Sun J. Yang X. (2022b). Colorimetric assay for acetylcholinesterase activity and inhibitor screening based on metal-organic framework nanosheets. Anal. Chem. 94 (47), 16345–16352. 10.1021/acs.analchem.2c03290 36444539

[B28] Wang H. Qian T. Zhao Y. Zhuo Y. Wu C. Osakada T. (2023). A tool kit of highly selective and sensitive genetically encoded neuropeptide sensors. Science 382 (6672), eabq8173. 10.1126/science.abq8173 37972184 PMC11205257

[B29] Xu T. Li S. Li A. J. Zhao J. Sakamuru S. Huang W. (2023). Identification of potent and selective acetylcholinesterase/butyrylcholinesterase inhibitors by virtual screening. J. Chem. Inf. Model 63 (8), 2321–2330. 10.1021/acs.jcim.3c00230 37011147 PMC10688023

[B30] Zavala-Ocampo L. M. Aguirre-Hernández E. López-Camacho P. Y. Cárdenas-Vázquez R. Dorazco-González A. Basurto-Islas G. (2022). Acetylcholinesterase inhibition and antioxidant activity properties of Petiveria alliacea L. J. Ethnopharmacol. 292, 115239. 10.1016/j.jep.2022.115239 35358623

[B31] Zhang B. Wang Y. Wu D. Zhao Q. Chen Y. Li Y. (2024). Fluorescent assay for acetylcholinesterase activity and inhibitor screening based on lanthanide organic/inorganic hybrid materials. Anal. Methods 16 (2), 314–321. 10.1039/d3ay01925j 38116865

[B32] Zhao X. Hu Q. Wang X. Li C. Chen X. Zhao D. (2024). Dual-target inhibitors based on acetylcholinesterase: novel agents for alzheimer's disease. Eur. J. Med. Chem. 279, 116810. 10.1016/j.ejmech.2024.116810 39243456

